# An Ancient Baboon Genome Demonstrates Long-Term Population Continuity in Southern Africa

**DOI:** 10.1093/gbe/evaa019

**Published:** 2020-02-05

**Authors:** Iain Mathieson, Federico Abascal, Lasse Vinner, Pontus Skoglund, Cristina Pomilla, Peter Mitchell, Charles Arthur, Deepti Gurdasani, Eske Willerslev, Manj S Sandhu, Genevieve Dewar

**Affiliations:** e1 Department of Genetics, Perelman School of Medicine, University of Pennsylvania; e2 Wellcome Sanger Institute, Hinxton, United Kingdom; e3 Centre for GeoGenetics, University of Copenhagen, Denmark; e4 Francis Crick Institute, London, United Kingdom; e5 School of Archaeology, University of Oxford, United Kingdom; e6 School of Geography, Archaeology and Environmental Studies, University of the Witwatersrand, Braamfontein, South Africa; e7 William Harvey Research Institute, Queen Mary’s University of London, United Kingdom; e8 Department of Zoology, University of Cambridge, United Kingdom; e9 The Danish Institute for Advanced Study, University of Southern Denmark, Odense, Denmark; e10 Department of Medicine, University of Cambridge, United Kingdom; e11 Department of Anthropology, University of Toronto Scarborough, Toronto, Ontario, Canada; e12 Omnigen Biodata Ltd., Cambridge, United Kingdom

**Keywords:** ancient DNA, baboons, demography

## Abstract

Baboons are one of the most abundant large nonhuman primates and are widely studied in biomedical, behavioral, and anthropological research. Despite this, our knowledge of their evolutionary and demographic history remains incomplete. Here, we report a 0.9-fold coverage genome sequence from a 5800-year-old baboon from the site of Ha Makotoko in Lesotho. The ancient baboon is closely related to present-day *Papio ursinus* individuals from southern Africa—indicating a high degree of continuity in the southern African baboon population. This level of population continuity is rare in recent human populations but may provide a good model for the evolution of *Homo* and other large primates over similar timespans in structured populations throughout Africa.

## Introduction

Baboons (genus *Papio*) are Old World Monkeys, widely distributed throughout Africa and the Arabian Peninsula. The six extant species of baboon occupy largely independent geographic ranges (Jolly 1993; Zinner et al. 2013) but readily hybridize in contact regions (Nagel 1973; Samuels and Altmann 1986; Jolly 1993; Jolly et al. 2011). The oldest splits among them date to 1.5–2 Ma, between Northern (*Papio* *hamadryas*, *Papio* *anubis*, and *Papio* *papio*) and Southern (*Papio* *ursinus* and *Papio* *cynocephalus*) clades (Zinner, Groeneveld, et al. 2009; Zinner et al. 2013; Rogers et al. 2019). The southernmost species (*P. ursinus*) has two deeply diverged subspecies (*ursinus* and *grisepes*), whose history and distribution may have been shaped by historical changes in range driven by aridification cycles (Sithaldeen et al. 2009; Sithaldeen et al. 2015). Thus, the boundary between the *P. ursinus* subspecies, as well as between *P. ursinus* and other species, may have shifted over time. Here, we test this by analyzing the genome of a 5800-year-old baboon from close to the present-day *ursinus/grisepes* contact zone. As well as illuminating this phylogeographic question, our results show more broadly the usefulness of ancient DNA for understanding the history, evolution and paleoenvironmental context of African primates.

## Results

**Fig. 1.**
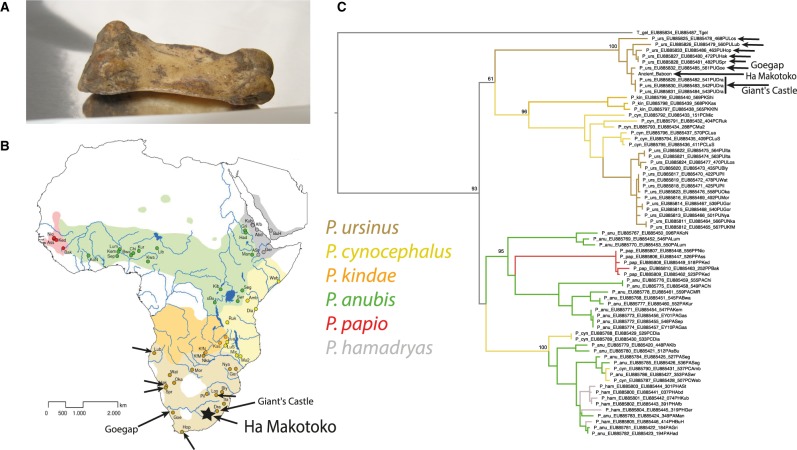
—Mitochondrial genome analysis. (*A*) Image of the ancient baboon phalanx. (*B*) Location of ancient baboon and geographic distribution of baboon species. Map: modified from Zinner, Groeneveld, et al. (2009) (CC BY). (*C*) Phylogenetic tree of the ancient baboon and present-day baboons using partial mitochondrial sequences from Zinner, Groeneveld, et al. (2009). The locations of the two most closely related mitochondrial genomes (U13/14) are indicated with labeled arrows in *B* and *C*, and those in the other southern *ursinus* haplogroups U10,11,12 and 15 are indicated with unlabeled arrows.

We extracted and sequenced DNA from a baboon proximal phalanx excavated at the archaeological site of Ha Makotoko, western Lesotho, and directly dated to ∼5,800 calBP (fig. 1*A* and *B*, supplementary table 1 and methods, Supplementary Material online). We generated, sequenced and aligned 13 libraries (supplementary table 2, Supplementary Material online) to the Panu_2.0 (*P. anubis*) nuclear genome, and to the *P. ursinus* mitochondrial genome. For these libraries, we estimated an average endogenous DNA content of 8.5% and obtained a total mean mapped autosomal coverage of 0.93× and mitochondrial coverage of 36.6×. The Panu_2.0 reference genome does not contain a Y chromosome, but comparison of coverage on the X chromosome (0.47) to the autosomes (mean 0.93, range 0.84–1.04) indicates that the phalanx belonged to a male. Fragment lengths (supplementary fig. 1, Supplementary Material online) and damage patterns are consistent with authentic ancient DNA (Dabney et al. 2013) with C>T transitions at 5′ ends affecting ∼15% of bases in the last position (supplementary fig. 2, Supplementary Material online). Low mitochondrial (1.3%) and X chromosome (0.47%) consensus mismatch at nonreference, nondamage sites indicates that contamination (from other baboons) is low. We restricted our analysis to reads with evidence of cytosine deamination, characteristic of authentic ancient DNA (Skoglund et al. 2014), and find results consistent with the unrestricted data (supplementary table 3, Supplementary Material online).

We compared the ancient baboon with mitochondrial data from 66 present-day baboons (Zinner, Groeneveld, et al. 2009), including Guinea (*P. papio*), olive (*P. anubis*), hamadryas (*P. hamadryas*), Kinda (*P. kindae*), yellow (*P. cynocephalus*), and chacma (*P. ursinus*) baboons. We included geladas (*Theropithecus gelada*) as an outgroup. These publicly available data include the complete coding sequence (CDS) of *CYTB*, part of the CDS of *NADH5* and the complete tRNA-His, tRNA-Ser, and tRNA-Leu sequences. The resulting tree (fig. 1*C*) shows that the ancient baboon has haplogroup U13/U14 and clusters with southern *P. ursinus* (i.e., Cape chacma, *P. ursinus ursinus*). Ha Makotoko is at the eastern end of the range of this subspecies, which extends to the south and west of the Kalahari Desert (Sithaldeen et al. 2015). The most closely related specimens come from the Giant’s Castle Game Reserve and the Goegap Nature Reserve (fig. 1*A*). We also compared the complete ancient mitochondrial genome (36× coverage) with complete mitochondrial genomes from 10 present-day baboons (Zinner et al. 2013), and to the 16 present-day baboons from the baboon genome project diversity panel (Rogers et al. 2019), confirming that it is closely related to present-day southern *P. ursinus* (supplementary fig. 3, Supplementary Material online).

**Fig. 2.**
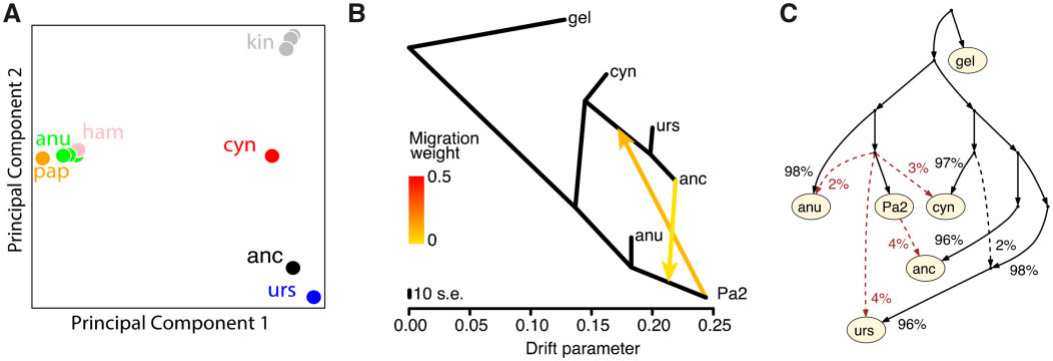
—Autosomal genome analysis. (*A*) First two principal components of genome-wide data. (*B*) TreeMix (Pickrell and Pritchard 2012) analysis of southern clade baboons with one northern clade representative (*Papio anubis*) and the papAnu2 (Pa2) reference genome with two migration edges (worst outlier 3.9 S.E). (*C*) An admixture graph that is consistent with the data (worst *D*-statistic *Z* score 0.4) of southern clade baboons with one northern clade representative (*P. anubis*) and the papAnu 2 reference genome. Other graphs may be equally consistent. Apparent gene flow (dashed red lines) between the Pa2 lineage and other samples likely reflects the effect of reference mapping bias. *Abbreviations*: anc, ancient baboon; anu, *P. anubis*; cyn, *Papio cynocephalus*; gel, *Theropithecus gelada*; ham, *Papio hamadryas*; kin, *Papio kindae*; pap, *Papio papio*; Pa2, papAnu2 reference genome.

Next, we analyzed the autosomal genome together with 14 present-day baboons sequenced as part of the baboon genome project diversity panel (Rogers et al. 2019). Heterozygosity at sites polymorphic in *P. cynocephalus* is similar in the ancient baboon (8.5%) to present-day *P. ursinus* (8.7%), suggesting a relatively constant level of genetic diversity. In principal component analysis (PCA), the ancient baboon falls closest to the two *P. ursinus* individuals (fig. 2*A*). D statistics (Patterson et al. 2012) suggest that the ancient baboon might carry some ancestry related to Northern clade subspecies such as *P. anubis*. In particular, D(*T. gelada*, *P. anubis*, *P. ursinus*, Ancient) has a *Z* score of 12.1 suggesting that the ancient baboon shares significant drift with *P. anubis* to the exclusion of *P. ursinus*. However, this is also consistent with differential attraction to the reference genome (generated from a *P. anubis* individual)—a common source of bias in ancient DNA studies (Cahill et al. 2018; Gunther and Nettelblad 2019; Sheng et al. 2019). This interpretation is supported by the *D* statistic D(*T. gelada*, Ancient, *P. anubis*, papAnu2) that has a *Z* score of 18.6 indicating that the ancient baboon shares more drift with the reference than other *P. anubis* individuals.

To further investigate the effect of reference bias, we constructed admixture graphs using TreeMix (Pickrell and Pritchard 2012) and qpGraph (Patterson et al. 2012) (fig. 2B and C, supplementary fig. 4 and methods, Supplementary Material online). TreeMix finds evidence of “gene flow”—in reality the effect of reference bias—between the Ancient lineage and the reference genome (fig. 2*B*). Similarly, qpGraph shows that the observed *D* statistics can be explained with small amounts of gene flow from the reference into other samples (fig. 2*C*). The qpGraph model suggests that, relative to the ancient baboon, present-day *P. ursinus* may carry a small amount (2%) of ancestry related to *P. cynocephalus*. However, we do not detect this signal using ADMIXTURE (Alexander et al. 2009) (supplementary fig. 5, Supplementary Material online) or *f*_3_ statistics (Patterson et al. 2012) (*f*_3_(*P. ursinus*; Ancient; *P. cynocephalus*); *Z* = 15.9) so it may be an artefact of the graph fitting or reflect structure within the *P. ursinus* population rather than admixture.

The Y chromosomal *TSPY* locus can distinguish between subspecies (Tosi et al. 2003; Jolly et al. 2011). We aligned reads to the *T. gelada* TSPY sequence (Tosi et al. 2003) and compared with seven reported sequences (Tosi et al. 2003; Zinner, Arnold, et al. 2009; Jolly et al. 2011) (supplementary table 4, Supplementary Material online). High coverage at this locus (because of multiple *TSPY* copies) confirms the male sex determination. The ancient baboon haplotype appears consistent with that reported for *P. ursinus griseipes* in the *ursinus*/*kindae* hybrid zone in Zambia (Jolly et al. 2011). At least one other *P. ursinus ursinus* individual from Southern Africa (Zinner, Arnold, et al. 2009) has a different haplotype (supplementary table 4, Supplementary Material online), raising the possibility of discordant mitochondrial and Y chromosome phylogenies, consistent with female philopatry and male dispersal in *P. ursinus* (Kopp et al. 2014).

We investigated the metrics of the phalanx (supplementary fig. 6, Supplementary Material online). The maximum length and medio-lateral midshaft breadth, at 26.3 and 7.1 mm, respectively are within the range of variation of an identified male *P. ursinus* (UCT/87/09) (*n* = 7, mean = 28.3 and 6.3 mm, SD = 5.1 and 0.8 mm) and a larger sample of *n* = 16 males represented by one third phalanx per individual (mean = 28.5 and 7.2 mm, SD = 2.4 and 0.7 mm) (Vernon 2013). However, the medio-lateral breadth at the base (the proximal joint) is 12.5 mm larger and outside the range of variation of both comparative samples (mean = 10.5 and 10.5 mm, SD = 0.9 and 0.8 mm). This suggests that it was more robust than a modern animal. Because the genetic data show that it is not a hybrid, this could reflect the effect of altitude (Bergman 1847; Sayers 2014). Carbon and nitrogen isotope values (δ^13^C = –16.99 ± 0.19‰ and δ^15^N = 4.46 ± 0.18‰ and a C:N ratio of 3.3) reflect a predominantly C_3_ diet, typical of *P. ursinus*, although the low value may reflect a diet more heavily focused on browse or fleshy fruits. The nitrogen values reflect low trophic level species and a largely vegetarian diet.

## Discussion

Our data demonstrate that *P. ursinus ursinus* persisted in the foothills of the Maloti-Drakensberg Mountains throughout the past 6,000 years. This is despite the fact that paleoenvironmental proxies predict drier conditions across the summer rainfall zone which may, for example, have limited human occupation across all of southeastern Southern Africa from 6.0 to 3.5 ka (Stewart and Mitchell 2018). It remains to be seen how far back in time this continuity extends, but more ancient genomes would address that question. Additional nuclear genomes from ancient and present-day baboons will allow us to estimate the extent and timing of gene flow between the *ursinus* and *grisepes* subspecies.

These data demonstrate that it is possible to extract and sequence high quality ancient genomes from southern Africa. This 0.9× genome is ∼3,500 years older than the oldest human shotgun genome from the region (Schlebusch et al. 2017) and demonstrates that it should be possible to obtain much older human genomes, but also genomes from the different baboon species and other nonhuman primates. In particular, the temporal resolution provided by ancient DNA allows precise comparisons with paleoclimate data, allowing tests of specific hypotheses about the relationship between climatic variation and phylogeography. Although the vast majority of effort in ancient DNA is geared toward humans or domesticated species, this study underscores the utility of ancient DNA for understanding the history and evolution of nonhuman species under natural conditions.

## Materials and Methods

### Data Processing and Quality Control

All ancient DNA work was performed in the aDNA clean lab facility of GeoGenetics, Copenhagen. From bone material obtained from the center of the phalanx, we extracted DNA (Allentoft et al. 2015) for 13 libraries, 9 of which were treated with the Uracil-Specific Excision Reagent (USER). aDNA libraries were prepared according to Meyer and Kircher (2010), using the modification described by Allentoft et al. (2015). The libraries were 75-bp paired-end sequenced on the Illumina platform HiSeq-2000 v4. Sequence reads were processed with AdapterRemoval v2.1.7 (Schubert et al. 2016) to: Remove adaptor remnants and low-quality ends (“Ns” and bases with quality < 20); merge read pairs when mate-pairs overlap at least 11 bp; and discard reads shorter than 30 bp. Parameters were “- -collapse - -minalignmentlength 11 - -minlength 30 - -trimqualities - -minquality 20 - -trimns - -qualitybase 33 - -qualitymax 42 - -mm 3.”

We aligned reads to the *P. anubis* (papAnu2) reference sequence (Rogers et al. 2019) using the *aln* algorithm of *bwa* v0.7.12 (Li and Durbin 2009), disabling seeding (-l 10000). We removed duplicates from each library BAM file using Picard tools v1.127 (http://broadinstitute.github.io/picard) and merged libraries using Samtools v1.2 (Li et al. 2009). Finally, we ran *GATK*’s *RealignerTargetCreator* and *IndelRealigner* v3.4.0 (McKenna et al. 2010) to identify potential indels and realign reads around them. The ancient baboon’s mitochondria clustered with *P. ursinus*, so to produce a more accurate whole-mitochondrial sequence, we replaced the reference *P. anubis* mitochondrial sequence with the complete mitochondrial sequence of *P. ursinus* from Zinner et al. (2013) (GenBank accession JX946205.2). We removed unplaced contigs and scaffolds.

We assessed the authenticity of the ancient DNA by confirming damage patterns characteristic of ancient samples, that is, DNA fragmentation and increased C>T transitions at the 5′ ends of DNA molecules. We used *bamdamage* (v20140602) from the *bammds* package (Malaspinas et al. 2014). The distribution of read lengths shows a peak around 40 bp. C>T transitions show high rates at 5′ ends, affecting 15% of cytosines (supplementary fig. 1, Supplementary Material online), consistent with the presence of authentic ancient DNA. For some analyses, we restricted to reads with evidence of damage using *pmdtools* (Skoglund et al. 2014) with the option --threshold = 3.

We tested for contamination by counting the proportion of reads that mapped to the mitochondria and did not match the majority call at sites where the majority call was nonreference. Across all sites we found that 3.6% of reads did not match, and at sites where a mismatch could not be the result of deamination, 1.3% of reads did not match. Because it is possible that potential contaminants share nonreference variants with the ancient individual, this is not a direct estimate of contamination, but nevertheless supports the authenticity of the data. We repeated the same analysis for the X chromosome, finding that 0.63% of all sites and 0.47% of nondeamination sites did not match.

### Mitochondrial Analysis

To call the mitochondrial genome of our ancient baboon we skipped the removal of nonuniquely aligned reads, as NUMTs in the nuclear genome result in missed coverage in the mitochondrial genome. We required each site to be covered by 10 or more reads with base qualities > 30 and that at least 80% consensus. For the partial mitochondrial genomes of Zinner, Groeneveld, et al. (2009) we aligned the data using Mafft v7.305b (Katoh and Standley 2013) and built a tree using *Phyml* v3.0 (Guindon et al. 2010) with a TN93+G+I+F model. For the complete mitochondrial genomes from Zinner et al. (2013) and Rogers et al. (2019), we aligned the whole-mitochondrial sequences with MUSCLE (Edgar 2004; Madeira et al. 2019), and estimated the maximum clade credibility tree using Beast2 (Bouckaert et al. 2019) with a GTR model.

### Autosomal Analysis

We generated pseudohaploid calls by picking a random base from all reads covering each site in the genome. We obtained baboon genome project diversity panel SNP calls from Rogers et al. (2019), merged with the pseudohaploid ancient calls, and restricted to transversions that were polymorphic in present-day baboons for all analysis. We ran TreeMix v1.13 (Pickrell and Pritchard 2012) with the “-root” option to use *T. gelada* as an outgroup, and the option “-noss” to turn off sample size correction. We ran qpGraph v6065 (Patterson et al. 2012), starting with the tree inferred by TreeMix and manually adding admixture edges until the absolute value of the worst *D* statistic *Z* score was <3.

We estimated conditional nucleotide diversity (CND) by restricting to sites that were polymorphic in a single *P. cynocephalus* individual, and counted how many were heterozygous in present-day *P. ursinus*. For the ancient baboon, we counted total *n_i_* and alternative *k_i_* allele counts at each SNP *i*, restricted to the *N* SNPs where *n_i_* > 1 and then estimated $\text{CND}=(2/N)\sum_{i=1}^{N}\left\{\left[n_{i}^{2}-k_{i}^{2}-{\left(n_{i}-k_{i}\right)}^{2}]/{[n}_{i}(n_{i}-1)\right]\right\}$. We averaged the results obtained from ascertaining sites in each of the two *P. cynocephalus* individuals, which were very similar.

To analyze *TSPY* we aligned reads from the ancient baboon that had not aligned to the reference to the *T. gelada TSPY* sequence; GenBank: AF284278.2 (Tosi et al. 2003). We also obtained the *P. hamadryas* sequence from the same reference, and four other partial sequences (Zinner, Arnold, et al. 2009) which we aligned to the *T. gelada* sequence to identify differences.

## Supplementary Material

Supplementary data are available at *Genome Biology and Evolution* online.

## Supplementary Material

evaa019_Supplementary_Data

## Notes

**Data deposition:** Sequence read data for the ancient baboon have been deposited at the European Nucleotide Archive under the accession ERP119178.

## References

[evaa019-B1] Alexander DH Novembre J Lange K. 2009 Fast model-based estimation of ancestry in unrelated individuals. Genome Res. 19(9):1655–1664. 19648217 10.1101/gr.094052.109 PMC2752134

[evaa019-B2] Allentoft ME, et al 2015 Population genomics of Bronze Age Eurasia. Nature 522(7555):167–172. 26062507 10.1038/nature14507

[evaa019-B3] Bergman C. 1847 Über die Verhältnisse der Wärmeökonomie der Thiere zu ihrer Grösse. Göttinger Studien. 3:595–708.

[evaa019-B4] Bouckaert R, et al 2019 BEAST 2.5: an advanced software platform for Bayesian evolutionary analysis. PLoS Comput Biol. 15(4):e1006650. 30958812 10.1371/journal.pcbi.1006650 PMC6472827

[evaa019-B5] Cahill JA, et al 2018 Genomic evidence of widespread admixture from polar bears into brown bears during the last ice age. Mol Biol Evol. 35(5):1120–1129. 29471451 10.1093/molbev/msy018

[evaa019-B6] Dabney J Meyer M Paabo S. 2013 Ancient DNA damage. Cold Spring Harb Perspect Biol. 5:a012567. 10.1101/cshperspect.a012567 PMC3685887 23729639

[evaa019-B7] Edgar RC. 2004 MUSCLE: multiple sequence alignment with high accuracy and high throughput. Nucleic Acids Res. 32(5):1792–1797. 15034147 10.1093/nar/gkh340 PMC390337

[evaa019-B8] Guindon S, et al 2010 New algorithms and methods to estimate maximum-likelihood phylogenies: assessing the performance of PhyML 3.0. Syst Biol. 59(3):307–321. 20525638 10.1093/sysbio/syq010

[evaa019-B9] Gunther T Nettelblad C. 2019 The presence and impact of reference bias on population genomic studies of prehistoric human populations. PLoS Genet. 15:e1008302. 31348818 10.1371/journal.pgen.1008302 PMC6685638

[evaa019-B10] Jolly CJ. 1993 Species, subspecies and baboon systematics In: Kimbel W Martin L, editors. Species, species concepts and primate evolution. New York: Wiley p. 67–107.

[evaa019-B11] Jolly CJ Burrell AS Phillips-Conroy JE Bergey C Rogers J. 2011 Kinda baboons (*Papio kindae*) and grayfoot chacma baboons (*P. ursinus griseipes*) hybridize in the Kafue river valley, Zambia. Am J Primatol. 73(3):291–303. 21274900 10.1002/ajp.20896

[evaa019-B12] Katoh K Standley DM. 2013 MAFFT multiple sequence alignment software version 7: improvements in performance and usability. Mol Biol Evol. 30(4):772–780. 23329690 10.1093/molbev/mst010 PMC3603318

[evaa019-B13] Kopp GH, et al 2014 The influence of social systems on patterns of mitochondrial DNA variation in baboons. Int J Primatol. 35(1):210–225. 24523566 10.1007/s10764-013-9725-5 PMC3915079

[evaa019-B14] Li H Durbin R. 2009 Fast and accurate short read alignment with Burrows–Wheeler transform. Bioinformatics 25(14):1754–1760. 19451168 10.1093/bioinformatics/btp324 PMC2705234

[evaa019-B15] Li H, et al 2009 The sequence alignment/map format and SAMtools. Bioinformatics 25(16):2078–2079 19505943 10.1093/bioinformatics/btp352 PMC2723002

[evaa019-B16] Madeira F, et al 2019 The EMBL-EBI search and sequence analysis tools APIs in 2019. Nucleic Acids Res. 47(W1):W636–W641. 10.1093/nar/gkz268 PMC6602479 30976793

[evaa019-B17] Malaspinas AS, et al 2014 bammds: a tool for assessing the ancestry of low-depth whole-genome data using multidimensional scaling (MDS). Bioinformatics 30(20):2962–2964. 24974206 10.1093/bioinformatics/btu410 PMC4184259

[evaa019-B18] McKenna A, et al 2010 The Genome Analysis Toolkit: a MapReduce framework for analyzing next-generation DNA sequencing data. Genome Res. 20(9):1297–1303. 20644199 10.1101/gr.107524.110 PMC2928508

[evaa019-B19] Meyer M Kircher M. 2010 Illumina sequencing library preparation for highly multiplexed target capture and sequencing. Cold Spring Harb Protoc. 2010(6):pdb.prot5448. 10.1101/pdb.prot5448 20516186

[evaa019-B20] Nagel U. 1973 A comparison of anubis baboons, hamadryas baboons and their hybrids at a species border in Ethiopia. Folia Primatol. 19(2–3):104–165. 4201907 10.1159/000155536

[evaa019-B21] Patterson N, et al 2012 Ancient admixture in human history. Genetics 192(3):1065–1093. 22960212 10.1534/genetics.112.145037 PMC3522152

[evaa019-B22] Pickrell JK Pritchard JK. 2012 Inference of population splits and mixtures from genome-wide allele frequency data. PLoS Genet. 8(11):e1002967. 23166502 10.1371/journal.pgen.1002967 PMC3499260

[evaa019-B23] Rogers J, et al 2019 The comparative genomics and complex population history of *Papio* baboons. Sci Adv. 5(1):eaau6947. 30854422 10.1126/sciadv.aau6947 PMC6401983

[evaa019-B24] Samuels A Altmann J. 1986 Immigration of a *Papio anubis* male into a group of *Papio cynocephalus* baboons and evidence for an anubis-cynocephalus hybrid zone in Amboseli. Int J Primatol. 7(2):131–138.

[evaa019-B25] Sayers K, 2014 High altitude primates, extreme primates, and anthropological primatology: or, there is more to human evolution than tool use, culture, or African apes In: Grow N, editors. High altitude primates. Developments in primatology: progress and prospects. New York: Springer.

[evaa019-B26] Schlebusch CM, et al 2017 Southern African ancient genomes estimate modern human divergence to 350,000–260,000 years ago. Science 358(6363):652–655. 28971970 10.1126/science.aao6266

[evaa019-B27] Schubert M Lindgreen S Orlando L. 2016 AdapterRemoval v2: rapid adapter trimming, identification, and read merging. BMC Res Notes. 9:88. 26868221 10.1186/s13104-016-1900-2 PMC4751634

[evaa019-B28] Sheng GL, et al 2019 Paleogenome reveals genetic contribution of extinct giant panda to extant populations. Curr Biol. 29(10):1695–1700.e1696. 31080081 10.1016/j.cub.2019.04.021

[evaa019-B29] Sithaldeen R Ackermann RR Bishop JM. 2015 Pleistocene aridification cycles shaped the contemporary genetic architecture of Southern African baboons. PLoS One 10(5):e0123207. 25970269 10.1371/journal.pone.0123207 PMC4430493

[evaa019-B30] Sithaldeen R Bishop JM Ackermann RR. 2009 Mitochondrial DNA analysis reveals Plio-Pleistocene diversification within the chacma baboon. Mol Phylogenet Evol. 53(3):1042–1048. 19665055 10.1016/j.ympev.2009.07.038

[evaa019-B31] Skoglund P, et al 2014 Separating endogenous ancient DNA from modern day contamination in a Siberian Neandertal. Proc Natl Acad Sci USA. 111(6):2229–2234. 24469802 10.1073/pnas.1318934111 PMC3926038

[evaa019-B32] Stewart BA Mitchell PJ. 2018 Late Quaternary palaeoclimates and human–environment dynamics of the Maloti-Drakensberg region, southern Africa. Quat Sci Rev. 196:1–20.

[evaa019-B33] Tosi AJ Disotell TR Morales JC Melnick DJ. 2003 Cercopithecine Y-chromosome data provide a test of competing morphological evolutionary hypotheses. Mol Phylogenet Evol. 27(3):510–521. 12742755 10.1016/s1055-7903(03)00024-1

[evaa019-B34] Vernon DS. 2013. A morphometric analysis of the phalanges and a fragmentary first metatarsal from the Drimolen hominin site [MPhil. thesis]. South Africa: University of Johannesburg.

[evaa019-B35] Zinner D Arnold ML Roos C. 2009 Is the new primate genus *Rungwecebus* a baboon? PLoS One 4(3):e4859. 19295908 10.1371/journal.pone.0004859 PMC2654078

[evaa019-B36] Zinner D Groeneveld LF Keller C Roos C. 2009 Mitochondrial phylogeography of baboons (*Papio* spp.): indication for introgressive hybridization? BMC Evol Biol. 9(1):83. 19389236 10.1186/1471-2148-9-83 PMC2681462

[evaa019-B37] Zinner D Wertheimer J Liedigk R Groeneveld LF Roos C. 2013 Baboon phylogeny as inferred from complete mitochondrial genomes. Am J Phys Anthropol. 150(1):133–140. 23180628 10.1002/ajpa.22185 PMC3572579

