# Supplementary material for: An Ancient Baboon Genome Demonstrates Long-Term Population Continuity in Southern Africa
Source: Genome Biol Evol. 2020 Feb 5;12(4):407–12. doi: 10.1093/gbe/evaa019 (PMC7197492; doi:10.1093/gbe/evaa019)
Supplement: evaa019_Supplementary_Data [file evaa019_supplementary_data.pdf]

Supplementary Information

Archaeological information

Ha Makotoko was a large (820 m^2) rock shelter above the Phuthiatsana River, western Lesotho. It was first excavated in 1989, and then again in 2010 as part of mitigation efforts in advance of the Metolong Dam, which drowned the site in 2014. Excavations showed that it was occupied by Middle Stone Age populations during Marine Isotope Stage (MIS) 3 and then by makers of successive Later Stone Age (LSA) traditions late in MIS 2 and again in the early Holocene, after which a thick body of culturally sterile silt and sand was deposited within the site (Mitchell 1993; Mitchell and Arthur 2014). Much more recent LSA occupation was represented by stone artefacts at and immediately below the surface that nevertheless lacked a well-defined archaeological context, and by rock paintings dated to the last 1000 years on the shelter's walls (Bonneau, et al. 2017). Local farmers then sporadically used the site to house livestock during the nineteenth and twentieth centuries.

The baboon phalanx discussed here was found in Context 001 of Square 64,50 in the surface sediments of Ha Makotoko Trench 61, located in the centre of the rock shelter some 5m in front of its rear wall. This context comprised loose surface dust and fine grayish-brown sandy silt with caprine and bovine dung, including clearly recognizable pellets as well as animal hair. Later Stone Age artefacts and other remains from Context 001 are *ex situ* and consist of both recent (≤1 ka) and early Holocene-aged material. None of the other 1595 identified bones from the Trench 61 deposits, which together represent 22 different species, were of baboon. A worn first molar of *P. ursinus* was, however, recovered from the Phase 6 early Holocene deposits in Trench 54 and likely dates to c. 10600-10300 cal. BP (Mitchell and Arthur 2014).

Bonneau A, Pearce D, Mitchell P, Staff R, Arthur C, Mallen L, Brock F, Higham T. 2017. The earliest directly dated rock paintings from southern Africa: new AMS radiocarbon dates. Antiquity 91:322-333.
Mitchell P. 1993. Archaeological investigations at two Lesotho rock-shelters: terminal Pleistocene/early Holocene assemblages from Ha Makotoko and Ntloana Tsoana. Proceedings of the Prehistoric Society 59:39-60.
Mitchell P, Arthur C. 2014. Ha Makotoko: Later Stone Age occupation across the Pleistocene/Holocene transition in western Lesotho. Journal of African Archaeology 12:205-232.

**Lab Number**	AAR-24006
**Collagen Yield**	0.153
**^{14}C age**	5059 ± 30 BP
**pMC**	53.27 ± 0.2
**δ^{13}C (CF-CN)**	-16.99 ± 0.19
**δ^{15}N (CF-CN)**	4.46 ± 0.18
**C:N ratio (TCD)**	3.283 ± 0.007
**Carbon fraction (TCD)**	44.165 ± 0.007
**Nitrogen fraction (TCD)**	15.695 ± 0.007
**Calibration and correction**	SHCal13 (Atmospheric)
**Calibrated Age**	68.2% probability<br>3931BC (29.8%) 3876BC<br>3806BC (27.0%) 3761BC<br>3742BC (11.4%) 3715BC<br>95.4% probability<br>3944BC (39.3%) 3854BC<br>3848BC (56.1%) 3707BC

Supplementary Table 1. ^{14}C dating information

Library	USER treated	Number of read pairs	Mapped reads	Uniquely mapped reads	Endogenous content %
LIB_AAG_ZA6266349	Yes	38,574,600	11,376,512	8,071,090	10.46
LIB_AAG_ZA6266351	Yes	20,218,569	7,526,488	4,609,442	11.40
LIB_AAG_ZA6266352	Yes	26,613,547	8,390,930	3,922,658	7.37
LIB_AAG_ZA6266368	No	19,234,827	5,286,787	3,365,619	8.75
LIB_AAG_ZA6266369	No	26,075,216	7,135,234	4,527,627	8.68
LIB_AAG_ZA6266370	No	27,454,825	11,626,213	6,852,905	12.48
LIB_AAG_ZA6266371	No	23,400,267	9,427,584	4,186,824	8.95
LIB_AAG_ZA6266386	Yes	28,651,953	3,666,601	1,920,124	3.35
LIB_AAG_ZA6266387	Yes	28,889,145	6,484,945	4,780,663	8.27
LIB_AAG_ZA6266388	Yes	27,163,266	8,097,680	5,014,113	9.23
LIB_AAG_ZA6266389	Yes	20,434,321	5,302,755	2,543,108	6.22
LIB_EGAN00001358735	Yes	18,849,240	8,886,300	2,345,962	6.22
LIB_EGAN00001358736	Yes	11,520,248	5,450,253	1,474,511	6.40
**Total**	9/13	317,080,024	98,658,282	53,614,646	8.45

Supplementary Table 2. Libraries constructed and library-specific statistics.

X	D	Z	BABA	ABBA	N Sites
P. anubis	0.0262	1.756	1553	1474	1,300,725
P. cynocephalus	-0.0254	-1.793	1597	1680	1,298,789
P. hamadryas	0.0126	0.79	1517	1479	1,297,872
P. kindae	-0.0184	-1.385	1607	1667	1,300,143
P. papio	0.0289	1.725	1505	1420	1,296,793
P. ursinus	-0.0404	-3.346	2561	2776	1,297,345
H. sapiens (hg19)	-0.0342	-1.523	894	957	947,739

Supplementary Table 3. D statistics of the form D(*T. gelada*, X, Ancient, Ancient damage restricted), comparing the full ancient data to the damaged restricted ancient data. Largely nonsignificant values indicate that contamination from other baboon subspecies, or from humans, is low. A significant D statistic when X=*P. ursinus* may indicate a low level of recent *P. ursinus* contamination.

	gel [T3]	ancient	ham [T3]	kin [J11]	urs [J11]	Urs [Z9]	pap [Z9]	cyn [Z9]	anu [Z9]
78	T	C	C	T	C	-	-	-	-
193	C	G	G	-	-	G	G	G	G
265	A	C	A	A	C	A	A	A	A
273	G	A	A	-	-	A	A	A	A
294	G	T	T	-	-	T	T	T	T
361	T	T	T	-	-	C	T	T	T
499	T	T	T	-	-	T	C	T	C
635	T	G	G	-	-	G	G	G	G
795	C	T	T	-	-	T	T	T	T
1185	G	A	A	-	-	A	A	A	A
1407	C	T	T	-	-	T	T	T	T
1434	T	T	T	G?	A?	T	T	T	T
1567	C	T	T	-	-	T	T	T	T
1709	C	G	G	-	-	-	-	-	-
1832	C	G	G	-	-	-	-	-	-
1840	C	G	G	-	-	-	-	-	-
1996	C	T	T	-	-	-	-	-	-
2024	T	G	G	-	-	-	-	-	-
2120	C	T	T	-	-	-	-	-	-
2203	G	A	A	-	-	-	-	-	-

Supplementary Table 4. Positions at the *TSPY* locus where one or more *Papio* subspecies differs from *T. gelada*. The ancient baboon has C's at positions 78 and 265, which matches the *P. ursinus* haplotype reported (n=7) in Jolly, et al. (2011) although position 265 does not match the *P. ursinus* haplotype reported (n=1) in Zinner, et al. (2009). At position 1434, the ancient baboon has a T, which does not match either the *P. ursinus* or *P. kindae* alleles in Jolly, et al. (2011), unless those alleles were reported on the negative strand, in which case the ancient baboon does match the *P. ursinus* allele. References in square brackets: T3 (Tosi, et al. 2003), J11 (Jolly, et al. 2011) and Z9 (Zinner, et al. 2009).

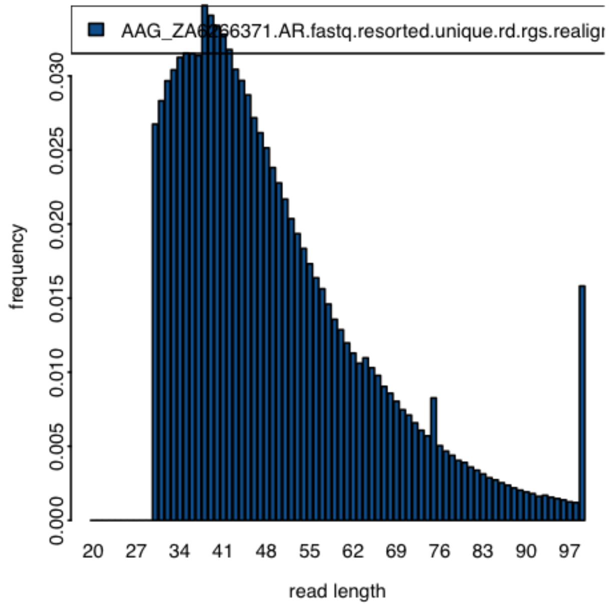

Supplementary Figure 1. Aligned read length distribution.

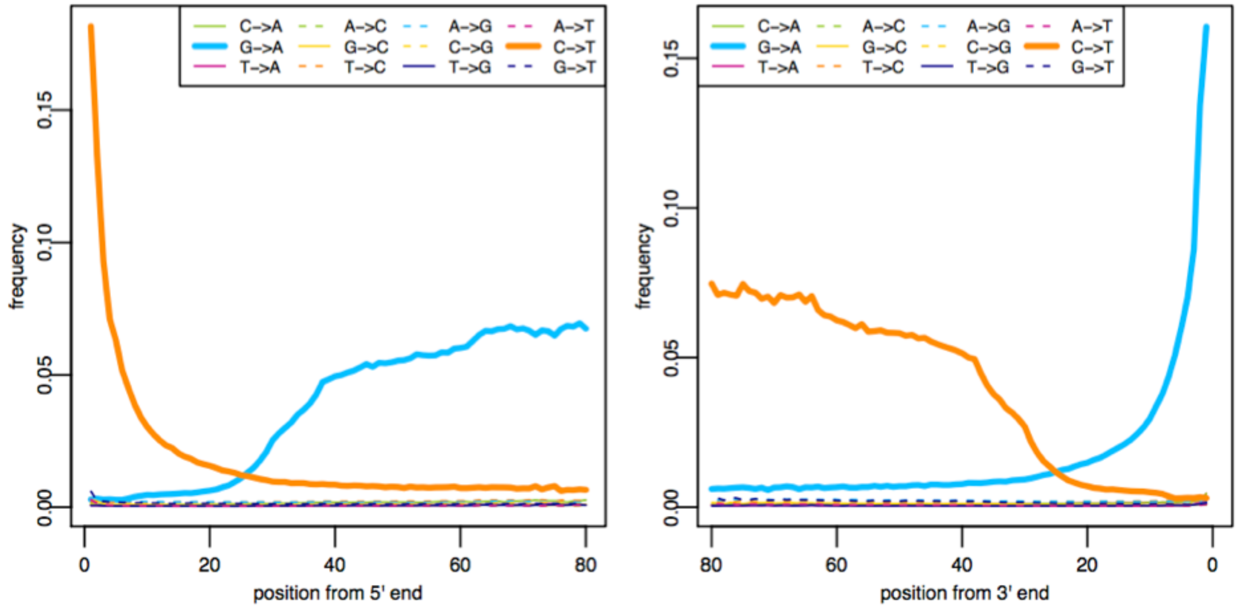

Supplementary Figure 2. Position-specific mismatch rates. Observed base-specific mismatch rates as a function of position of the base in the read.

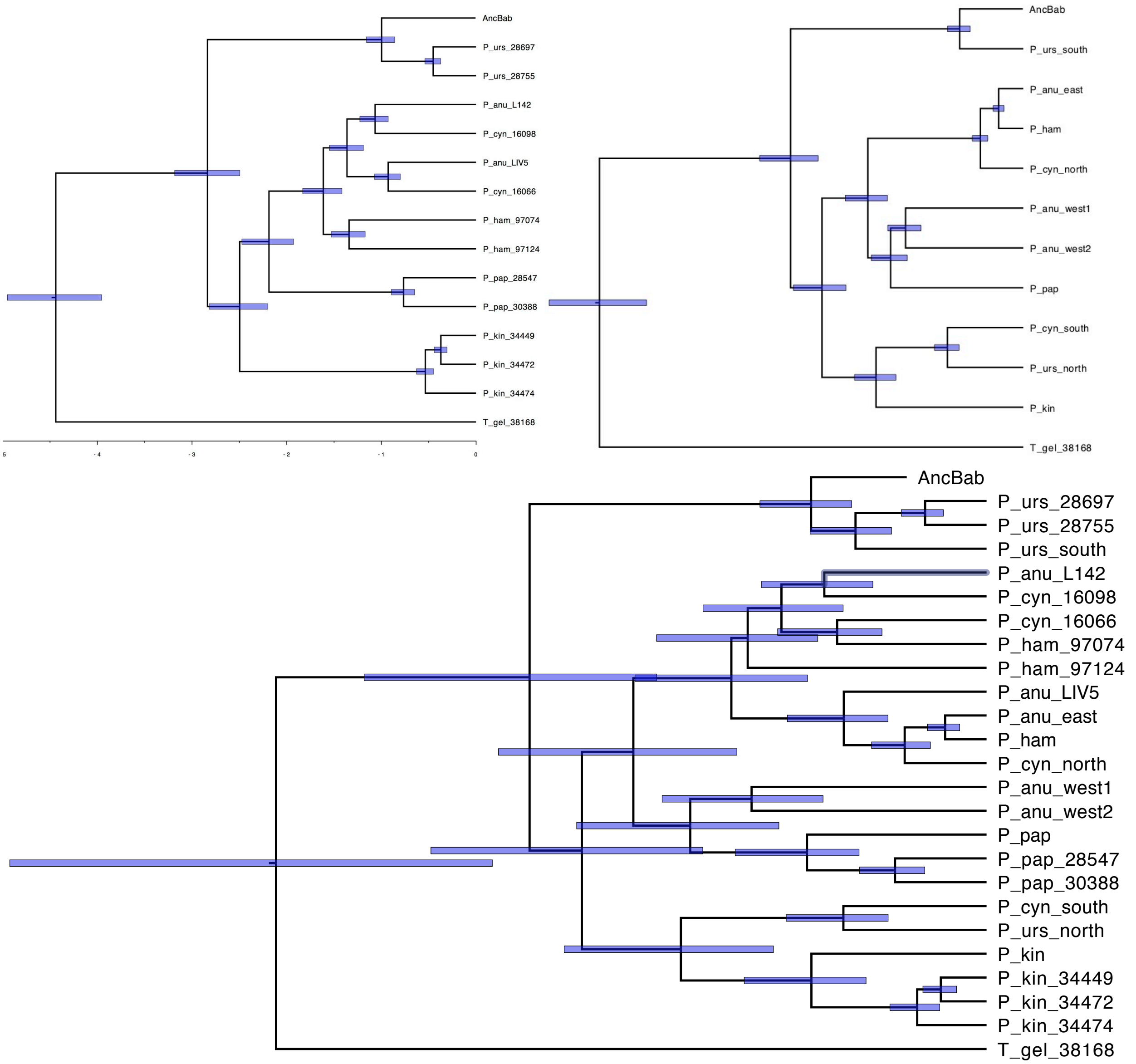

Supplementary Figure 3. Maximum clade credibility trees inferred from whole mitochondrial genomes. Bars show 90% highest posterior density node heights. **Upper Left**: sequences from Rogers, et al. (2019). **Upper Right**: sequences from Zinner, et al. (2013). **Lower**: Both datasets combined. Plotted using FigTree (http://tree.bio.ed.ac.uk/software/figtree/). Note that the two Rogers, et al. (2019) *P. ursinus* samples are from captive populations, so cannot be directly linked to a geographic area although their mitochondrial genomes, like that of the ancient individual, cluster with wild southern *P. ursinus* so they are very likely *P. ursinus ursinus*.

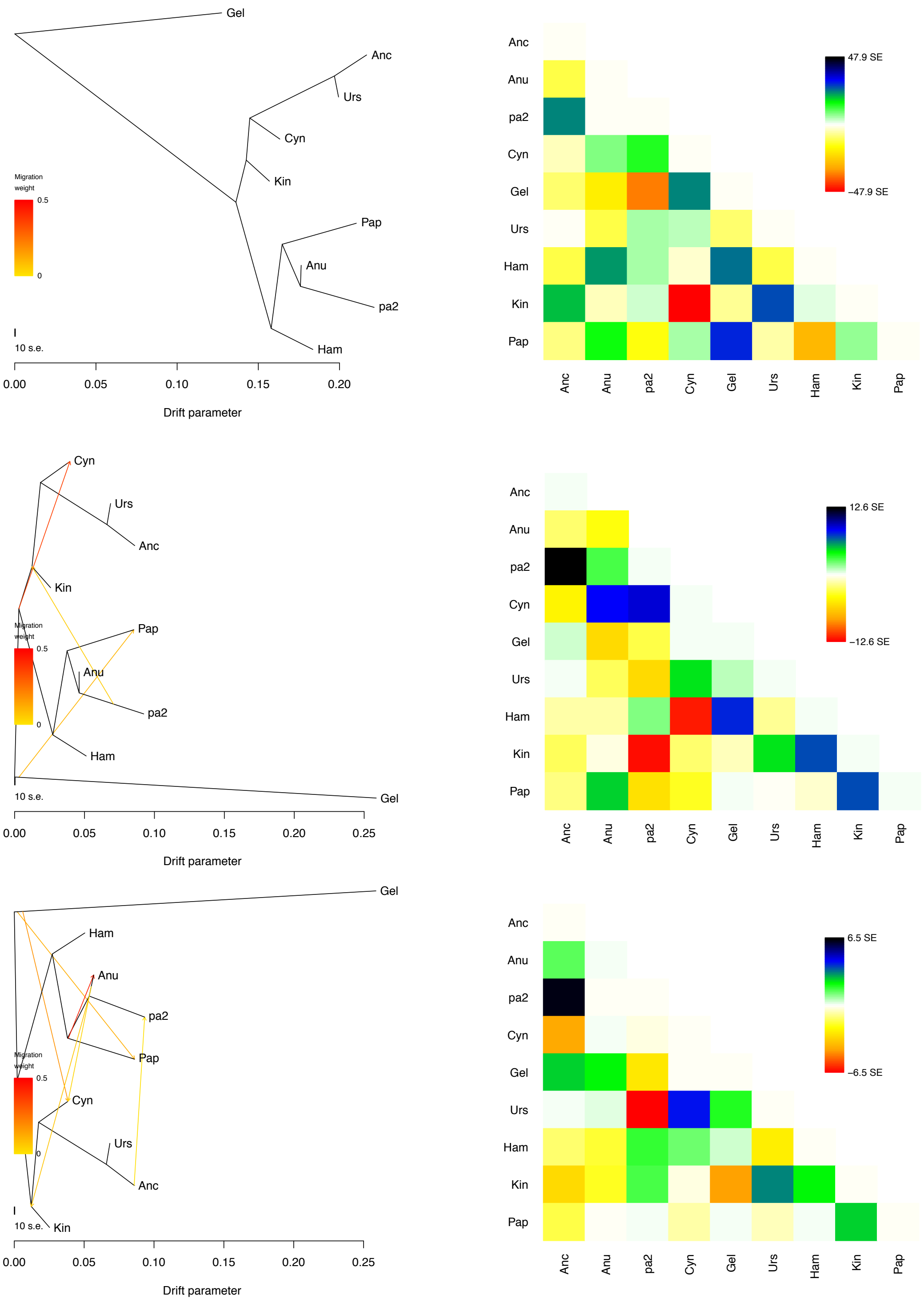

Supplementary Figure 4. Treemix models (left) and residuals (right) for all populations, with 0, 3 and 6 migration edges.

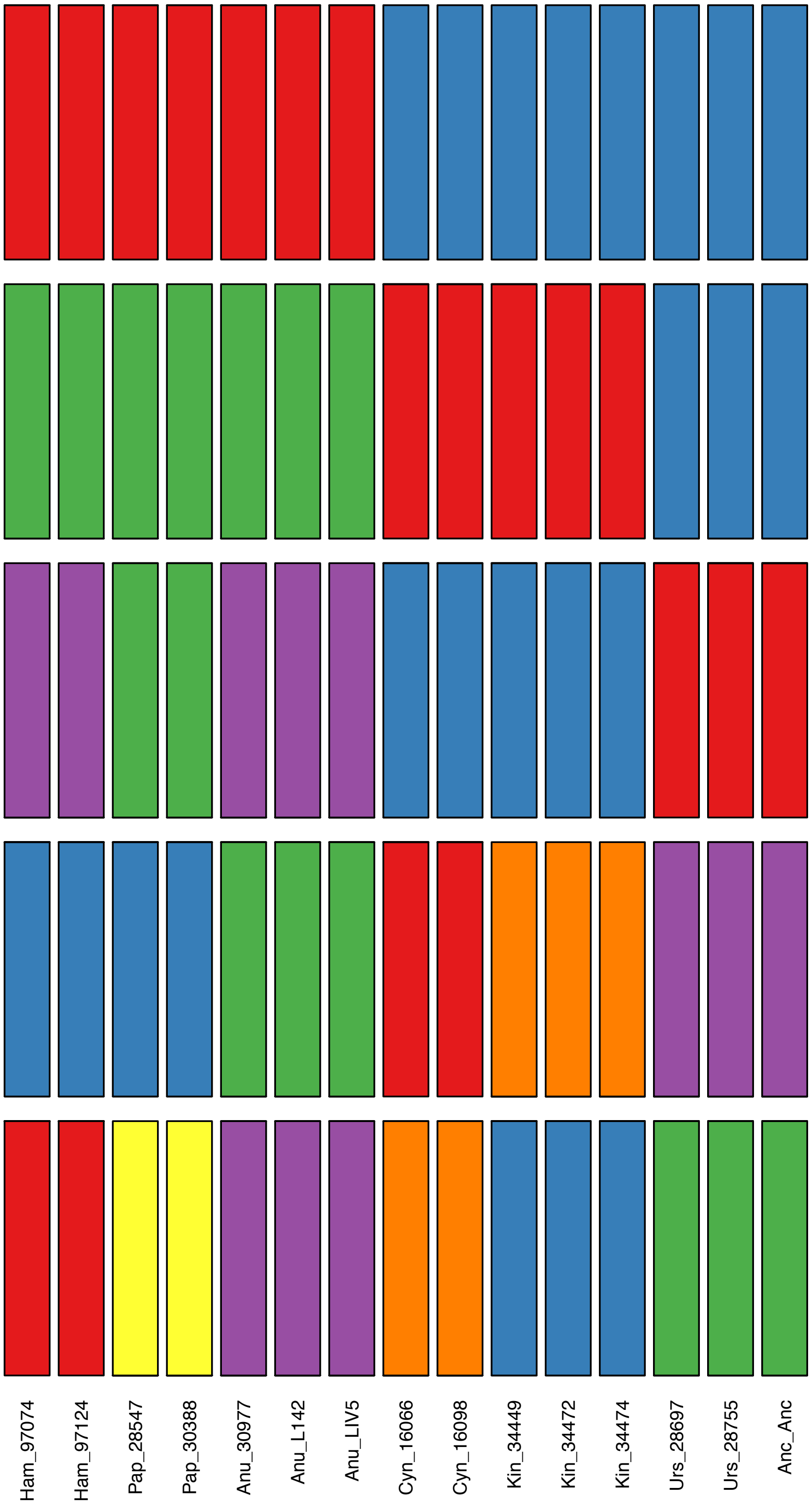

Supplementary Figure 5: ADMIXTURE analysis of the ancient baboon along with 14 present-day baboons from k=2 to 6. Present-day *P. ursinus* clusters with the ancient baboon and shows no sign of recent admixture from other species.

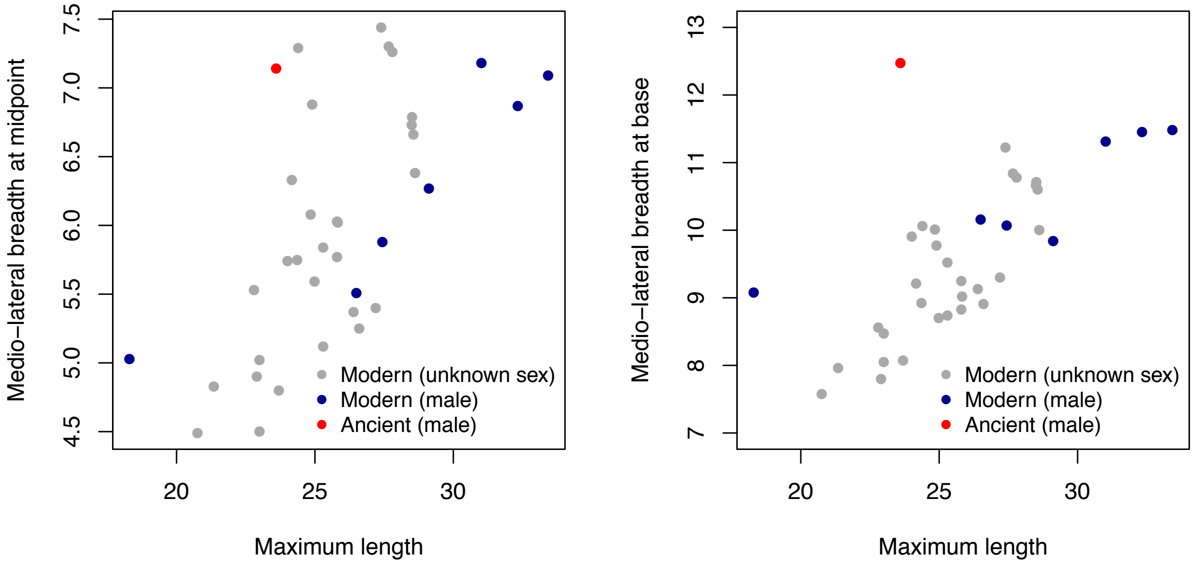

Supplementary Figure 6. Comparison of the size of the phalanx to proximal phalanges of present-day baboons (N=36 from 4 individuals). The length of the ancient baboon phalanx is within the range of present-day individuals but it is broader, particularly at the base.
